# Cultural intelligence and anxiety in English-mediated social interaction among Chinese international students in the UK: mediating roles of psychological resilience and fear of negative evaluation

**DOI:** 10.3389/fpsyg.2026.1802991

**Published:** 2026-04-10

**Authors:** Xiaoyun Liu, Ziqing Xu, Huilin Wang

**Affiliations:** 1 School of Literature and Journalism, Hunan First Normal University, Changsha, China; 2 Business School, Guangdong Ocean University, Yangjiang, China; 3 School of Business, Hunan University of Science and Technology, Xiangtan, China

**Keywords:** Chinese international students, cultural intelligence, fear of negative evaluation, psychological resilience, social interaction anxiety

## Abstract

**Introduction:**

English-mediated social interaction is a key component of second language (L2) learning and social adaptation among Chinese international students. In the United Kingdom, Chinese international students often face cultural adjustment challenges and social-evaluative concerns when using English, which may contribute to social interaction anxiety. This study examines the association between cultural intelligence and social interaction anxiety and explores the mediating roles of psychological resilience and fear of negative evaluation.

**Methods:**

Using a cross-sectional survey design, data were collected in December 2025 from 441 Chinese international students studying in the UK using an online questionnaire. Participants were recruited through convenience and snowball sampling. Structural equation modelling (SEM) was conducted using AMOS 26 to test the hypothesized associations and indirect pathways.

**Results:**

Cultural intelligence was positively associated with psychological resilience and negatively associated with fear of negative evaluation. Psychological resilience was negatively associated with both fear of negative evaluation and social interaction anxiety, whereas fear of negative evaluation was positively associated with social interaction anxiety. Bootstrapping analyses indicated a significant indirect association between cultural intelligence and social interaction anxiety through psychological resilience and fear of negative evaluation. The model explained 22% of the variance in psychological resilience, 30% of the variance in fear of negative evaluation, and 34% of the variance in social interaction anxiety.

**Discussion:**

The findings suggest that cultural intelligence may function as a potential psychological resource associated with lower social interaction anxiety in English-mediated contexts. However, because the data are cross-sectional, causal interpretations should be made with caution. The results underscore the importance of culturally informed and psychologically supportive practices in English-medium higher education and contribute to the literature on culture and L2 learning among Chinese international students.

## 1 Introduction

Over the past two decades, global higher education has undergone substantial transformation characterized by the rapid expansion of cross-border student mobility and the diversification of learning pathways. According to UNESCO (2025), approximately 6.9 million students were enrolled in tertiary education outside their home countries in 2023, a figure that has more than tripled since 2000. This sustained growth reflects not only the increasing demand for internationally recognized qualifications but also the globalization of academic labor markets and the internationalization strategies of universities (Tran et al., 2023; Glass and Cruz, 2023). As higher education becomes increasingly transnational, students are required to navigate unfamiliar academic conventions, diverse peer groups, and complex sociocultural expectations in host countries.

Within this global landscape, English has consolidated its position as the dominant medium of instruction and academic exchange, not only in Anglophone destinations such as the UK but also in many non-Anglophone contexts (Selvi et al., 2024; Sah, 2022). English-mediated communication therefore extends beyond formal classroom instruction and permeates everyday peer interaction, collaborative learning, and participation in campus life. Research in L2 acquisition consistently emphasizes that meaningful social interaction plays a crucial role in language development, as interaction provides opportunities for negotiation of meaning, feedback, and identity construction (Mifka-Profozic, 2023; Trofimovich et al., 2023). At the same time, successful engagement in such interaction is closely linked to broader indicators of sociocultural adaptation and psychological well-being among international students (Yu and Wright, 2024). Together, these trends underscore the importance of understanding how students experience and manage English-mediated social interaction in host environments.

Despite the opportunities afforded by international study, a substantial body of research indicates that L2 communication can be accompanied by heightened anxiety. MacIntyre and McGillivray (2023) conceptualized language anxiety as a situation-specific form of anxiety arising in contexts involving L2 use and demonstrated its detrimental effects on cognitive processing and performance. Subsequent research has shown that anxiety is particularly salient in spontaneous interpersonal interaction, where learners must respond in real time and may fear negative social judgment (Toyama and Yamazaki, 2022; Yılmaz and De Jong, 2024). Unlike structured examinations, social interaction involves dynamic, unpredictable exchanges, which can intensify self-consciousness and apprehension (Tavares, 2024). This issue may be especially relevant in the UK context, where Chinese international students encounter unfamiliar interaction norms, intensive English-medium environments, and culturally embedded evaluative expectations.

Within the UK, Chinese international students represent one of the largest international student populations (Zhu and Sharp, 2022). Although many achieve high academic results, qualitative and quantitative studies have documented challenges related to social integration, classroom participation, and informal interaction with host nationals (Yu and Moskal, 2019; Liu et al., 2022a). These challenges are often not solely attributable to linguistic proficiency but are intertwined with cultural differences in communication style, perceived expectations of participation, and concerns about social evaluation (Raja et al., 2021; Liu et al., 2025). Research further indicates that anxiety in L2 contexts can reduce willingness to communicate and limit engagement with local peers (Wei and Xu, 2022). Consequently, English-mediated social interaction anxiety may restrict opportunities for both linguistic development and meaningful intercultural contact. Taken together, these findings suggest that Chinese international students in the UK may face a particularly complex combination of intercultural demands and social-evaluative pressures in English-mediated interaction.

Existing scholarship has generated substantial insights into both L2 anxiety and cross-cultural adaptation. However, these two streams of research have largely evolved in parallel rather than in integration. Studies of L2 anxiety have primarily concentrated on classroom-based experiences, instructional dynamics, and performance-related stressors (Sparks and Patton, 2013; Welesilassie and Nikolov, 2024; Cheng and Sun, 2025). By contrast, research in cross-cultural psychology has focused more broadly on adaptation outcomes, including sociocultural adjustment, psychological well-being, and intercultural competence (Bierwiaczonek and Waldzus, 2016; Lo Presti et al., 2020; Li, 2020). As a result, comparatively less attention has been directed toward understanding how intercultural capabilities and individual psychological characteristics may jointly relate to anxiety experienced in everyday social interactions conducted through a L2. Within adjacent literatures, several constructs appear theoretically relevant to this study. Cultural intelligence has been conceptualized as a multidimensional capability that enables individuals to function effectively in culturally diverse environments (Yari et al., 2020; Wang and Goh, 2020) and has been associated with more positive cross-cultural adjustment outcomes (Ott and Michailova, 2018). Resilience research emphasizes individuals’ capacity to adapt constructively in the face of stress and adversity (Southwick et al., 2005). Fear of negative evaluation refers to the tendency to experience apprehension about being judged unfavorably by others (Carleton et al., 2007; Weeks and Howell, 2012) and has been consistently associated with heightened social anxiety in interpersonal contexts (Glory and Subekti, 2021). Although these constructs have been examined separately, less is known about how intercultural competence, adaptive psychological resources, and evaluative cognitive processes operate together in shaping anxiety during every day L2 social interaction, particularly outside classroom settings. This gap highlights the need for an integrative framework that captures the joint contributions of cultural intelligence, resilience, and fear of negative evaluation.

The present study addresses these gaps by examining how cultural intelligence relates to social interaction anxiety in English-mediated contexts among Chinese international students in the UK. Specifically, it investigates the association between cultural intelligence and social interaction anxiety in everyday interpersonal engagement beyond classroom settings, including informal academic communication and peer interactions in culturally diverse groups. It also examines whether psychological resilience and fear of negative evaluation serve as mediating mechanisms, integrating both adaptive psychological resources and evaluative cognitive processes. By doing so, this study extends prior research on L2 anxiety, which has often focused on classroom performance, self-efficacy, or emotion regulation, by highlighting the combined roles of intercultural competence, resilience, and cognitive appraisal in everyday social interaction anxiety. The UK context provides a particularly relevant setting for this investigation, as Chinese students must navigate both intercultural demands and social-evaluative pressures in academic and social interactions, making it possible to observe how personal resources and cognitive factors jointly influence anxiety. By combining these perspectives, the study aims to provide a more nuanced, empirically grounded understanding of the psychological and intercultural factors that affect second-language social interaction, offering insights that can inform both theory and institutional support practices.

The paper is organized as follows. Section 2 presents the hypotheses and conceptual framework. Section 3 describes the methods employed for data collection and analysis. Section 4 reports the results of the data analysis and evaluates the proposed hypotheses. Section 5 offers a discussion, highlighting theoretical contributions, practical implications, and limitations, and provides suggestions for future research. Finally, Section 6 concludes the paper.

## 2 Literature review and hypothesis development

### 2.1 Conservation of resources theory

Conservation of resources theory provides a comprehensive framework for understanding stress and emotional responses in demanding environments (Hobfoll, 2001). The theory posits that individuals are motivated to obtain, retain, and protect resources that they value, and that psychological stress arises when resources are threatened, lost, or insufficiently gained following investment. Resources encompass personal characteristics, energies, conditions, and objects that are either inherently valuable or instrumental in achieving valued goals (Hobfoll et al., 2016). A key assumption is that resource loss is disproportionately more salient than resource gain (Sonnentag and Meier, 2024), which explains why perceived threats to competence, social standing, or psychological stability can trigger substantial stress even without actual loss. The theory further proposes resource loss and gain spirals, where individuals with abundant resources are better positioned to acquire additional resources and buffer against environmental demands, whereas those experiencing depletion are more vulnerable to further losses and negative outcomes (Hobfoll, 2011). Importantly, conservation of resources theory emphasizes that these processes are dynamic and context-sensitive (Zhu et al., 2025). Within intercultural and educational settings, individual psychological and social capabilities can be conceptualized as critical resources that influence adaptation outcomes. In the present study, cultural intelligence can be regarded as a personal and social competence resource that enables effective functioning in culturally diverse interactions. Psychological resilience constitutes a psychological energy resource that supports coping with environmental demands and mitigates stress responses. Fear of negative evaluation reflects perceived threats to social and self-related resources, signaling potential loss in social standing or self-esteem during evaluative interactions.

Integrating these constructs within the conservation of resources framework highlights the mechanisms through which personal resources influence anxiety in intercultural social contexts. Cultural intelligence and psychological resilience can be conceptualized as resource reserves that buffer against perceived threats, whereas fear of negative evaluation represents cognitive appraisal of potential resource loss. Within this framework, the interplay between available resources and perceived threat can account for individual differences in social interaction anxiety among Chinese international students. This approach allows for the systematic operationalization of constructs as specific resource types, providing a theoretically coherent basis for hypothesizing the mediating roles of resilience and evaluative concerns in English-mediated social interactions.

### 2.2 Hypothesis development

#### 2.2.1 Cultural intelligence, psychological resilience, and fear of negative evaluation

Cultural intelligence refers to an individual’s capability to function effectively in culturally diverse settings, encompassing cognitive, motivational, and behavioral dimensions (Van Dyne et al., 2012; Ng et al., 2012). Prior research has demonstrated that individuals with high cultural intelligence exhibit greater adaptability, emotional regulation, and cross-cultural effectiveness (Ng et al., 2022; Clark and Polesello, 2017). These adaptive capabilities suggest that cultural intelligence may serve as an important psychological resource in challenging and uncertain environments (Darvishmotevali et al., 2018).

From the perspective of Social Cognitive Theory, individuals’ beliefs about their capabilities influence how they perceive and respond to stressors (Benight and Bandura, 2004). The motivational and metacognitive components of cultural intelligence enhance individuals’ confidence in navigating unfamiliar cultural contexts, thereby strengthening self-efficacy in intercultural interactions (Egwuonwu et al., 2020). Higher intercultural self-efficacy, in turn, has been consistently linked to greater resilience when facing stress and adversity (Tomul et al., 2024). Furthermore, employees with high cultural intelligence are more likely to interpret cultural differences as learning opportunities rather than threats (Afsar et al., 2020). Such positive cognitive appraisal reduces perceived stress intensity and facilitates adaptive coping strategies, both of which are central components of psychological resilience.

Empirical evidence also supports a positive linkage between intercultural competence and resilience-related constructs. Studies have shown that individuals with higher cultural intelligence demonstrate better psychological adjustment, lower emotional exhaustion, and stronger recovery from cross-cultural strain (Chu and Zhu, 2023). In international work contexts, cultural intelligence has been found to enhance adaptability and persistence in the face of cultural challenges, which are core characteristics of resilience (Dolce et al., 2023). On this basis, the following hypotheses are advanced:

*Hypothesis 1 (H1)*: Cultural intelligence is positively associated with psychological resilience.

Research in second-language and intercultural communication indicates that individuals frequently encounter situations characterized by ambiguity, performance pressure, and potential social evaluation (Hu et al., 2021; Tian and McCafferty, 2022). Cognitive models of social anxiety posit that individuals’ perceptions of threat and their appraisal of coping resources shape evaluative concerns in social situations (Leigh and Clark, 2018; Toyama and Yamazaki, 2022). Specifically, when individuals perceive themselves as capable of interpreting social cues, anticipating others’ responses, and regulating their behavior, they are less likely to experience apprehension about potential negative judgment. Cultural intelligence, defined as the capability to function effectively across culturally diverse contexts (Wang and Goh, 2020; Van der Horst Catherine and Albertyn Ruth, 2018), provides individuals with metacognitive awareness, knowledge of cultural norms, motivational engagement, and behavioral adaptability. These capabilities enhance interpretive accuracy and confidence in intercultural interactions (Alexandra et al., 2021), which, based on cognitive appraisal theory, could theoretically reduce perceived social threat in situations involving evaluation by others. While empirical research has not yet directly examined the relationship between cultural intelligence and fear of negative evaluation, this theoretical reasoning suggests a plausible link. On this basis, the following hypotheses are advanced:

*Hypothesis 2 (H2)*: Cultural intelligence is negatively associated with fear of negative evaluation.

Psychological resilience refers to an individual’s capacity to maintain or regain adaptive psychological functioning in the face of stress and adversity (Sisto et al., 2019). In cross-cultural and second-language communication contexts, students frequently face challenges such as unfamiliar social norms, linguistic demands, and evaluative uncertainty, which may increase psychological strain (Tekel et al., 2025; Xu and Bt. Shapii, 2025). Resilience has been shown to enhance adaptive coping, facilitate emotion regulation, and promote persistence under stress (Egan et al., 2024; Kołodziej et al., 2025). Although few studies have directly examined the relationship between psychological resilience and fear of negative evaluation, prior research suggests that resilient individuals tend to experience lower psychological distress in challenging interpersonal contexts (Li and Yang, 2016; Kristiana et al., 2022). By promoting effective coping and sustaining psychological functioning, resilience may indirectly reduce the attentional and emotional focus on potential evaluative threats, thereby decreasing preoccupation with negative judgment. This reasoning is also consistent with Conservation of Resources theory, which suggests that individuals with more internal psychological resources are better able to buffer against stressors and maintain psychological well-being (Hobfoll et al., 2016). On this basis, the following hypotheses are advanced:

*Hypothesis 3 (H3)*: Psychological resilience is negatively associated with fear of negative evaluation.

#### 2.2.2 Psychological resilience, fear of negative evaluation, and social interaction anxiety

Social interaction anxiety refers to the distress and apprehension experienced during face-to-face interpersonal exchanges, particularly in situations involving perceived social scrutiny (Calleja-Núñez et al., 2023; Heimberg et al., 1992). Compared with broader social anxiety constructs, social interaction anxiety focuses on discomfort and tension arising in real-time social exchanges. In English-mediated academic environments, international students frequently encounter socially demanding interactions, such as group discussions, presentations, and informal peer communication, which may heighten vulnerability to social interaction anxiety (Szepesi et al., 2024; Song et al., 2026; Alasmari, 2023).

Accumulating empirical evidence demonstrates a robust negative association between psychological resilience and social anxiety. Zhao et al. (2025), in a study examining individuals with aphasia, found that psychological resilience was significantly negatively associated with social anxiety, with interpersonal relationship perception serving as a mediating mechanism. Similarly, Li and Zheng (2025) reported that trait resilience significantly predicted lower levels of social anxiety among college students, and this protective effect operated through adaptive emotion regulation and coping strategies. These findings suggest that psychological resilience functions as a protective psychological factor that mitigates vulnerability to socially evaluative distress across diverse populations. On this basis, the following hypotheses are advanced:

*Hypothesis 4 (H4)*: Psychological resilience is negatively associated with social interaction anxiety.

According to cognitive theories of social anxiety, fear of negative evaluation is considered a key cognitive mechanism underlying social anxiety and related distress in socially evaluative situations. Fear of negative evaluation refers to the tendency to experience apprehension and distress over the possibility of *being* judged unfavorably by others (Topel et al., 2024; Carleton et al., 2006). Empirical research indicates that fear of negative evaluation is strongly associated with social anxiety across diverse populations. Liu et al. (2022b) found that among Chinese adolescents, higher fear of negative evaluation was significantly linked to greater social anxiety symptoms. Similarly, Sigurvinsdottir et al. (2021) demonstrated in a virtual reality setting that individuals with higher fear of negative evaluation experienced greater distress during social performance tasks, indicating that evaluative concerns directly influence anxiety in real-time social interactions.

In second-language communication contexts, additional worries about language proficiency, communicative performance, or potential cultural misunderstandings may further heighten sensitivity to social evaluation, thereby increasing anxiety during face-to-face interactions (Zegrean, 2025; Zhang et al., 2025). These findings collectively suggest that individuals with higher fear of negative evaluation are more likely to experience elevated social interaction anxiety in English-mediated settings. On this basis, the following hypothesis is proposed:

*Hypothesis 5 (H5)*: Fear of negative evaluation is positively associated with social interaction anxiety.

#### 2.2.3 Mediation effects

In intercultural communication settings, individuals are required not only to interpret social cues accurately but also to regulate emotional responses to perceived evaluation and uncertainty (Dinu et al., 2025). Thus, understanding how intercultural capability translates into reduced anxiety requires attention to both adaptive psychological resources and evaluative cognitive processes. Psychological resilience represents an individual’s capacity to sustain adaptive functioning under stress (Denckla et al., 2020). Individuals with stronger resilience are more likely to maintain emotional stability and employ constructive coping strategies when encountering challenging social situations. From a conservation of resources perspective, resilience functions as a personal resource that buffers against perceived threat, reducing the likelihood that environmental demands translate into anxiety. Resilient individuals tend to perceive demanding interpersonal encounters as manageable rather than threatening, which may decrease their sensitivity to potential social evaluation (Maqsood et al., 2024). In this sense, resilience may shape how individuals interpret potential social judgment, influencing the degree to which they experience fear of negative evaluation (Breakwell, 2021). This conceptualization clearly distinguishes resilience as a resource-based capacity from anxiety, which is the emotional outcome of interest.

Fear of negative evaluation, in turn, reflects heightened sensitivity to possible social scrutiny. When individuals anticipate unfavorable judgment, they are more likely to engage in self-focused attention and threat-oriented interpretation during interpersonal exchanges, thereby increasing social interaction anxiety (Bates et al., 2024). Empirical research has shown that fear of negative evaluation is closely associated with social interaction anxiety and can function as a proximal predictor of anxious responses in real-time social settings (Spiroiu and Maranzan, 2025; Üstündağ et al., 2025). Theoretically, fear of negative evaluation represents a cognitive appraisal process that translates the availability of personal resources into emotional outcomes.

Taken together, cultural intelligence may first enhance psychological resilience by equipping individuals with adaptive coping strategies and confidence in intercultural contexts. Higher resilience, as a protective resource, reduces vulnerability to evaluative threat, thereby lowering fear of negative evaluation. In turn, reduced fear of negative evaluation diminishes social interaction anxiety. This sequential process is consistent with conservation of resources theory, which emphasizes that resources enable individuals to manage stressors, and with social-cognitive perspectives, which highlight the mediating role of cognitive appraisal in shaping emotional responses. On this basis, the following hypothesis is proposed:

*Hypothesis 6 (H6)*: Psychological resilience and fear of negative evaluation jointly mediate the relationship between cultural intelligence and social interaction anxiety.

**Figure 1:**
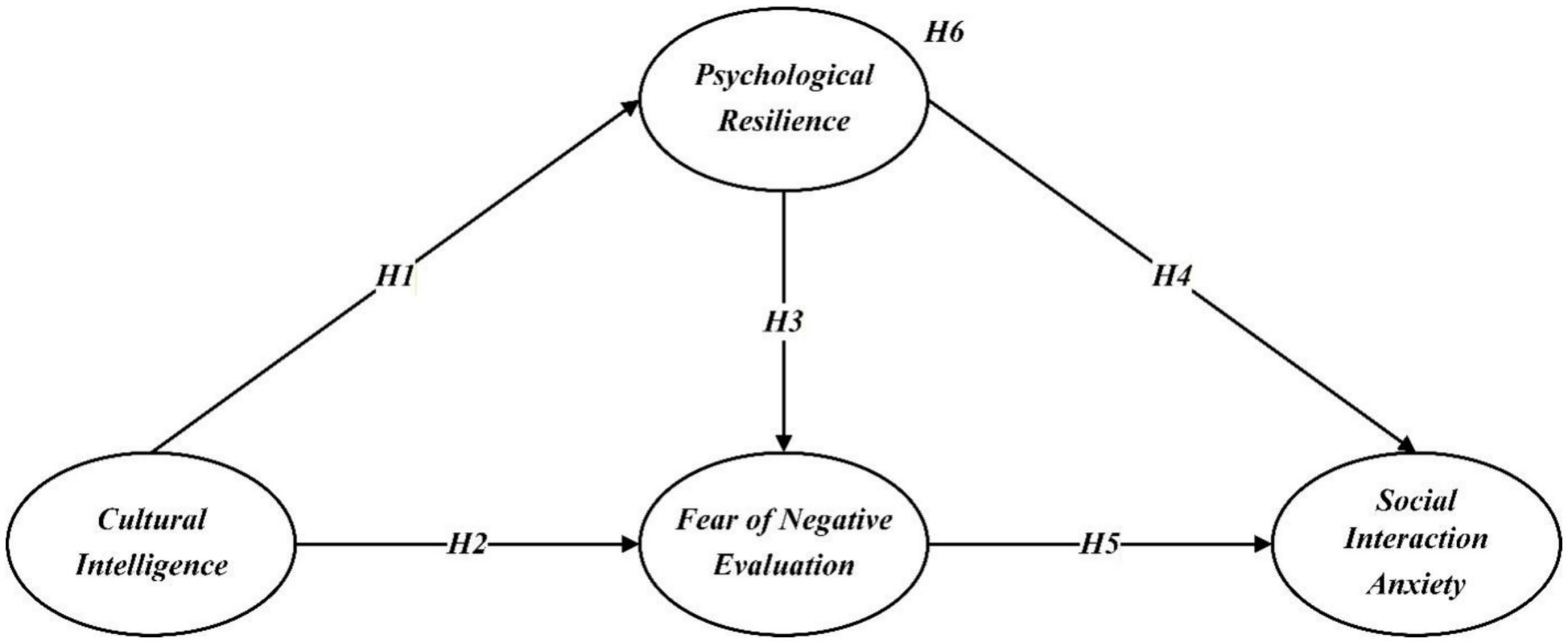
Hypothesis model.

Figure 1 summarizes the proposed hypotheses.

## 3 Methodology

### 3.1 Participants and procedures

Data were collected in December 2025 from Chinese international students studying in the UK. Participants were recruited using a combination of convenience sampling and snowball sampling. The survey link was distributed through Chinese international student online communities, including WeChat groups and student social media platforms commonly used by Chinese students in UK universities. Participants were also encouraged to share the survey link with other eligible students within their academic and social networks.

A total of 500 questionnaires were collected, after which data collection was discontinued. To improve data quality and verify whether respondents were located in the UK, the IP addresses of all responses were examined. Questionnaires were excluded if the IP address indicated that the respondent was located outside the United Kingdom. In addition, questionnaires with excessively short completion times were removed, as such responses may indicate insufficient engagement with the survey items. After the screening process, 441 valid questionnaires were retained for analysis. Detailed demographic information of the respondents, including gender, age, level of study, field of study, and length of study in the UK, was collected and is reported in Table 1.

**Table 1:** Demographic characteristics (*N* = 441).

Variable	Category	%
Gender	Male	45.6
	Female	54.4
Age (years)	18–20	26.5
	21–23	40.1
	24–26	21.8
	27 and above	11.6
Level of study	Undergraduate	66.0
	Master’s	26.1
	Doctoral	7.9
Field of study	Social Sciences / Business	34.7
	Science, Technology, Engineering, and Mathematics (STEM)	36.7
	Humanities and Arts	22.4
	Other	6.1
Length of study in the UK	Less than 1 year	29.9
	1–2 years	44.2
	2–4 years	19.7
	More than 4 years	6.1

As shown in Table 1, the sample consisted of 441 participants, with females accounting for a slightly higher proportion than males. Most participants were aged between 21 and 23 years, which is consistent with the typical age range of university students. The majority were undergraduates, reflecting the composition of the student population. Participants were drawn from a range of academic fields, with STEM and social sciences/business being the most represented. In terms of study experience in the UK, most participants had studied for one to 2 years.

### 3.2 Instruments

All measures were administered using an online questionnaire. Participants were instructed to respond to all items based on their experiences in English-mediated social interactions and daily academic and social life in the United Kingdom (e.g., in class, on campus, or in everyday situations). Unless otherwise stated, all items were rated on a five-point Likert scale ranging from 1 (strongly disagree) to 5 (strongly agree).

Cultural intelligence was measured using a shortened version of the Cultural Intelligence Scale developed by Thomas et al. (2015). The scale consists of 10 items assessing individuals’ cognitive, motivational, metacognitive, and behavioral capabilities in cross-cultural interactions. A sample item is: “I can change my behavior to suit different situations and people.” Higher scores indicate higher levels of cultural intelligence.

Psychological resilience was assessed using the Brief Resilience Scale (BRS-6) (Smith et al., 2008), which measures individuals’ ability to recover from stress and adversity. Participants were instructed to respond to the items based on their experiences of coping with challenges and stressors encountered during their study and daily life in the UK. A sample item is: “I tend to bounce back quickly after hard times.” Higher scores reflect greater psychological resilience.

Fear of negative evaluation was measured using the Brief Fear of Negative Evaluation Scale—Revised (BFNE-R) (Carleton et al., 2006). The scale includes eight positively worded items assessing concerns about being negatively evaluated by others. Participants responded to the items with reference to their experiences of interacting with others in English within academic and social contexts in the UK. A sample item is: “I worry about what kind of impression I make on people.” Higher scores represent stronger fear of negative evaluation.

Social interaction anxiety was assessed using the short form of the Social Interaction Anxiety Scale (SIAS-6) (Peters et al., 2012). Participants were instructed to respond to all items based on their experiences when interacting with others in English (e.g., in class, on campus, or in daily social situations) in the United Kingdom. A sample item is: “I have difficulty talking with other people.” Higher scores indicate higher levels of social interaction anxiety.

### 3.3 Data analysis

Data analyses were conducted using AMOS 26. A two-step analytical approach was adopted. First, confirmatory factor analysis (CFA) was performed to assess the measurement model, including construct reliability and validity. Second, structural equation modelling (SEM) was used to test the hypothesized relationships among cultural intelligence, psychological resilience, fear of negative evaluation, and social interaction anxiety. Indirect effects were examined using bootstrapping procedures.

To assess potential common method variance (CMV), Harman’s single-factor test was conducted using principal component analysis. The results showed that multiple factors with eigenvalues greater than 1 emerged, and the first unrotated factor accounted for 38.62% of the total variance, which is below the recommended threshold of 40%. These results suggest that common method variance is unlikely to be a serious concern in the present study.

## 4 Results

### 4.1 Measurement model

CFA was conducted to evaluate the reliability and validity of the measurement model. As shown in Table 2, all factor loadings were above the recommended threshold of 0.70, ranging from 0.749 to 0.815, indicating satisfactory item reliability.

**Table 2:** Reliability and convergent validity of the measurement model.

Items	Factor loadings	Cronbach’s *α*	CR	AVE
Cultural intelligence (CI)		0.940	0.939	0.608
CI1	0.780			
CI2	0.774			
CI3	0.783			
CI4	0.785			
CI5	0.795			
CI6	0.788			
CI7	0.749			
CI8	0.778			
CI9	0.769			
CI10	0.798			
Psychological resilience (PR)		0.903	0.903	0.608
PR1	0.777			
PR2	0.773			
PR3	0.793			
PR4	0.773			
PR5	0.786			
PR6	0.775			
*Fear of negative evaluation (FNE)*		0.930	0.930	0.625
FNE1	0.764			
FNE2	0.782			
FNE3	0.812			
FNE4	0.787			
FNE5	0.815			
FNE6	0.767			
FNE7	0.798			
FNE8	0.800			
Social interaction anxiety (SIA)		0.901	0.901	0.603
SIA1	0.792			
SIA2	0.759			
SIA3	0.800			
SIA4	0.775			
SIA5	0.752			
SIA6	0.778			

Internal consistency reliability was supported, with Cronbach’s alpha values ranging from 0.901 to 0.940 and composite reliability (CR) values ranging from 0.901 to 0.939, all exceeding the recommended criterion of 0.70. Convergent validity was also established, as the average variance extracted (AVE) values for all constructs ranged from 0.603 to 0.625, exceeding the suggested threshold of 0.50.

Discriminant validity was assessed using the Fornell–Larcker criterion. As presented in Table 3, the square roots of the AVE values for each construct were greater than the corresponding inter-construct correlations, providing evidence of adequate discriminant validity.

**Table 3:** Correlations and discriminant validity.

Construct	CI	PR	FNE	SIA
CI	(0.780)			
PR	0.429**	(0.780)		
FNE	−0.413**	−0.443**	(0.791)	
SIA	−0.424**	−0.459**	0.449**	(0.777)

The square roots of AVE are shown on the diagonal; off-diagonal elements are Pearson correlations among constructs. *p* < 0.01.

Additionally, discriminant validity was further assessed using the heterotrait–monotrait ratio (HTMT). All HTMT values were below the recommended threshold of 0.85, providing additional evidence of satisfactory discriminant validity.

Overall, these results indicate that the measurement model demonstrates satisfactory reliability and construct validity, supporting its suitability for subsequent structural model analysis.

### 4.2 Structural model

Structural equation modelling was conducted using AMOS 26 to examine the hypothesized associations among the study variables. The structural model demonstrated a good fit to the data: *χ^2^*/df = 1.13, RMR = 0.055, GFI = 0.938, AGFI = 0.927, NFI = 0.949, IFI = 0.994, TLI = 0.993, and CFI = 0.994, all of which met or exceeded commonly accepted criteria.

**Figure 2:**
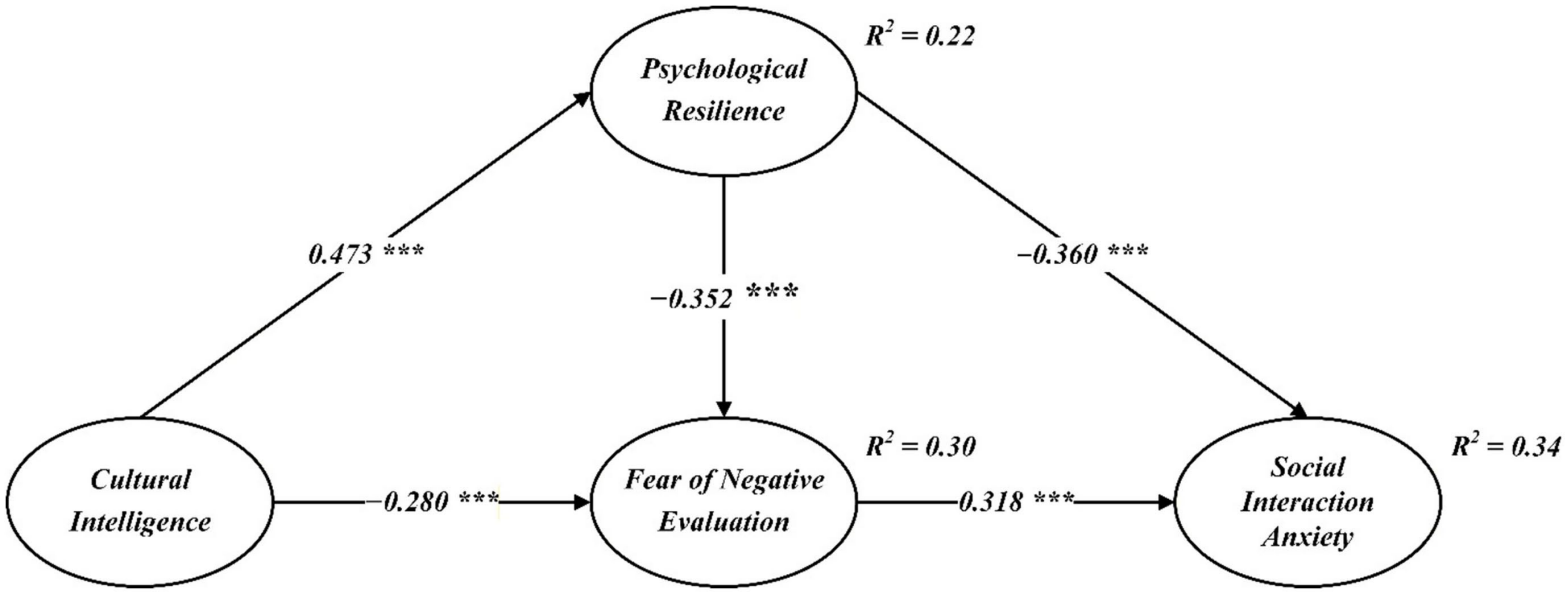
Structural model. *p* < 0.001.

As illustrated in Figure 2, cultural intelligence was positively associated with psychological resilience (*β* = 0.473, *p* < 0.001) and negatively associated with fear of negative evaluation (*β* = −0.280, *p* < 0.001). Psychological resilience was negatively associated with fear of negative evaluation (*β* = −0.352, *p* < 0.001) and social interaction anxiety (*β* = −0.360, *p* < 0.001). In addition, fear of negative evaluation was positively associated with social interaction anxiety (*β* = 0.318, *p* < 0.001). These results provide support for Hypotheses 1 to 5.

The model explained 22% of the variance in psychological resilience, 30% of the variance in fear of negative evaluation, and 34% of the variance in social interaction anxiety, indicating a moderate explanatory power of the proposed structural model.

This hypothesis was tested using a bootstrapping procedure. As shown in Table 4, the indirect effect of cultural intelligence on social interaction anxiety through psychological resilience and fear of negative evaluation was significant [standardized indirect effect = −0.313, SE = 0.033, 95% bias-corrected CI (−0.378, −0.251), *p* < 0.001]. Because no direct path from cultural intelligence to social interaction anxiety was specified in the structural model, the direct effect was constrained to zero, and the total effect is therefore fully represented by the indirect effect. Since the confidence interval did not include zero, Hypothesis 6 was supported.

**Table 4:** Mediation effects of cultural intelligence on social interaction anxiety.

Path	Point estimate	Product of coefficients		Bootstrapping		
				Bias-corrected 95% CI		Two-tailed significance
		SE	Z	Lower	Upper	
CI → SIA	−0.313	0.033	−9.485	−0.378	−0.251	*p* < 0.001

## 5 Discussion

### 5.1 Theoretical contributions

The present study offers several theoretical contributions to the literature on cultural intelligence, social interaction anxiety, and psychological processes in cross-cultural educational contexts.

First, this study extends research on cultural intelligence by examining its role in predicting social on anxiety in English-mediated interpersonal contexts. While prior research has predominantly linked cultural intelligence to cross-cultural adjustment, job performance, and intercultural effectiveness (Van Dyne et al., 2012; Ng et al., 2012), considerably less attention has been paid to its relevance for anxiety-related outcomes in everyday second-language interaction. By demonstrating that cultural intelligence is associated with lower levels of social interaction anxiety among Chinese international students in the UK, the present findings suggest a potential expansion of the scope of outcomes associated with intercultural capability. More importantly, the results indicate that cultural intelligence may function not only as a performance-oriented competence but also as a psychological resource that contributes to emotional regulation in socially evaluative settings.

Second, this study contributes to the literature on L2 anxiety and social interaction anxiety by shifting the analytical focus from classroom-bound performance contexts to naturally occurring interpersonal engagement in multicultural settings. Much of the L2 anxiety literature has concentrated on instructional environments and task-based language use (Cheng and Sun, 2025; Zabihi et al., 2021). In contrast, the present study situates anxiety within informal academic and peer interactions, providing a framework for understanding how linguistic competence, intercultural capability, and evaluative cognitive processes jointly shape social interaction anxiety in multicultural academic settings. This approach advances theory by conceptualizing social interaction anxiety as an interculturally situated phenomenon rather than solely a language proficiency issue.

Third, this study advances theoretical integration by bringing Conservation of Resources theory into dialogue with intercultural competence and social anxiety research. Although Conservation of Resources theory has been widely applied to occupational stress and coping (Hobfoll, 2001), its application to intercultural communication and second-language social interaction remains limited. By conceptualizing cultural intelligence and psychological resilience as personal resources and fear of negative evaluation as a cognitive indicator of perceived resource threat, the study provides a resource-based explanation of how intercultural capability may indirectly influence social interaction anxiety. The identification of a joint mediating pathway through psychological resilience and fear of negative evaluation deepens understanding of the psychological processes linking intercultural competence to emotional outcomes. In doing so, the study bridges previously separate theoretical traditions and offers a more integrated framework for explaining anxiety in culturally complex communication environments.

Overall, these contributions represent an incremental extension of existing theory, demonstrating that cultural intelligence influences not only cross-cultural adjustment but also emotional experiences in English-mediated social interaction. The study highlights the importance of considering both personal resources and evaluative cognitive processes when examining social interaction anxiety, providing a more nuanced, process-oriented perspective that can inform future research and theoretical development in cross-cultural psychology and L2 communication.

### 5.2 Practical implications

The present study offers several empirically grounded implications for supporting Chinese international students in English-medium higher education contexts in the UK, based on the demonstrated pathways linking cultural intelligence, psychological resilience, fear of negative evaluation, and social interaction anxiety.

First, fostering cultural intelligence can serve as a foundational strategy for mitigating social interaction anxiety. Institutions may implement targeted intercultural training programs that include orientation sessions, discipline-specific workshops, and peer-guided activities. These initiatives could focus on familiarizing students with UK academic norms, classroom participation expectations, and everyday social conventions. By enhancing students’ ability to interpret social cues accurately and navigate culturally diverse settings, such programs can indirectly strengthen psychological resilience and reduce anxiety during English-mediated interactions. Furthermore, embedding reflective exercises and scenario-based practice into these programs may allow students to consolidate learning and self-assess their intercultural competence.

Second, interventions aimed at enhancing psychological resilience can provide direct support for coping with social and academic stressors. Counseling services, stress management workshops, and group-based resilience training can equip students with strategies for emotional regulation and adaptive problem-solving in challenging intercultural interactions. Institutions may consider monitoring resilience outcomes through periodic self-report measures or reflective journals to assess intervention effectiveness. Strengthening resilience is particularly important in settings where students face continuous evaluative demands, such as oral presentations, group discussions, and peer collaborations.

Third, reducing fear of negative evaluation is critical for facilitating confident engagement in English-mediated social interaction. Pedagogical practices that normalize language-related challenges, emphasize formative feedback, and provide structured opportunities for participation can create a psychologically safe classroom environment. Peer mentoring, collaborative learning, and scaffolded group discussions may help students gradually build confidence, reducing self-focused attention and threat-oriented interpretations. Clear communication of assessment criteria and constructive feedback can further attenuate perceived evaluative threat.

Fourth, integrated support approaches that simultaneously address cultural, psychological, and social factors are likely to be most effective. Coordinating language support services with psychological counseling, intercultural workshops, and academic guidance ensures that interventions target both individual capabilities and environmental demands. For example, aligning intercultural training with classroom participation strategies and resilience-building exercises can reinforce learning outcomes and promote sustained reductions in social interaction anxiety.

Finally, while these recommendations are grounded in the current study’s findings, their implementation should be considered exploratory and context-sensitive. Institutions may pilot programs in selected departments or cohorts, collect feedback, and adjust interventions based on measurable indicators such as changes in students’ resilience scores, self-reported anxiety levels, and classroom participation frequency. This evidence-informed, iterative approach allows universities to balance feasibility, resource allocation, and potential impact while providing actionable guidance for reducing L2 social interaction anxiety in multicultural academic settings.

### 5.3 Limitations

Several limitations of this study should be acknowledged. First, the data were collected using a cross-sectional design, which limits the ability to draw causal inferences among cultural intelligence, psychological resilience, fear of negative evaluation, and social interaction anxiety. Although the proposed associations are theoretically grounded and empirically supported, future studies employing longitudinal or experimental designs would help clarify the temporal ordering of these relationships in L2 learning and cross-cultural adaptation processes.

Second, the sample consisted exclusively of Chinese international students studying in the UK. While this focus allows for a context-specific understanding of English-mediated social interaction in the UK, it may limit the generalizability of the findings to migrant students from other cultural backgrounds or to international students in different host countries. In addition, participants were recruited through convenience and snowball sampling, which may introduce self-selection bias, as students who chose to participate in the survey may differ systematically from those who did not. Future research could extend this model to more diverse migrant groups and adopt more systematic sampling strategies to improve representativeness.

Third, all variables in this study were measured using self-reported questionnaires, which may raise concerns regarding common method bias. Although Harman’s single-factor test suggested that common method variance was unlikely to be a serious concern, this statistical approach has limitations in fully ruling out common method bias, and the reliance on self-reported measures may still introduce shared method variance and contextual interpretation differences. Moreover, although established scales were used, measurement validity may be influenced by cultural and contextual factors when applied to Chinese international students in the UK. In addition, the study did not control for English language proficiency, which is a known predictor of L2 communication anxiety and may influence students’ social interaction experiences. Future research could employ multi-source data, time-lagged designs, and further examine the cross-cultural validity of these measurement instruments.

## 6 Conclusion

This study examined the associations between cultural intelligence and social interaction anxiety among Chinese international students studying in the United Kingdom, with psychological resilience and fear of negative evaluation as mediating mechanisms. The findings suggest that higher cultural intelligence is associated with lower levels of social interaction anxiety, and that this association operates through increased psychological resilience and reduced fear of negative evaluation. Psychological resilience and fear of negative evaluation jointly function as key psychological pathways linking cultural intelligence to social interaction anxiety in English-mediated social interaction contexts.

These findings contribute to the literature on intercultural communication and L2 social interaction by highlighting the psychological processes through which intercultural capability may relate to anxiety in multicultural academic environments. In particular, the results underscore the potential importance of psychological resilience and evaluative concerns in shaping international students’ experiences of L2-mediated social interaction.

## Data Availability

The raw data supporting the conclusions of this article will be made available by the authors, without undue reservation.
